# Prognoses Associated With Palliative Performance Scale Scores in Modern Palliative Care Practice

**DOI:** 10.1001/jamanetworkopen.2024.20472

**Published:** 2024-07-08

**Authors:** Kara E. Bischoff, Kanan Patel, W. John Boscardin, David L. O’Riordan, Steven Z. Pantilat, Alexander K. Smith

**Affiliations:** 1 Division of Palliative Medicine, Department of Medicine, University of California, San Francisco; 2 Division of Geriatrics, Department of Medicine, University of California, San Francisco

## Abstract

**Importance:**

The Palliative Performance Scale (PPS) is one of the most widely used prognostic tools for patients with serious illness. However, current prognostic estimates associated with PPS scores are based on data that are over a decade old.

**Objective:**

To generate updated prognostic estimates by PPS score, care setting, and illness category, and examine how well PPS predicts short- and longer-term survival.

**Design, Setting, and Participants:**

This prognostic study was conducted at a large academic medical center with robust inpatient and outpatient palliative care practices using electronic health record data linked with data from California Vital Records. Eligible participants included patients who received a palliative care consultation between January 1, 2018, and December 31, 2020. Data analysis was conducted from November 2022 to February 2024.

**Exposure:**

Palliative care consultation with a PPS score documented.

**Main Outcomes and Measures:**

The primary outcomes were predicted 1-, 6-, and 12-month mortality and median survival of patients by PPS score in the inpatient and outpatient settings, and performance of the PPS across a range of survival times. In subgroup analyses, mortality risk by PPS score was estimated in patients with cancer vs noncancer illnesses and those seen in-person vs by video telemedicine in the outpatient setting.

**Results:**

Overall, 4779 patients (mean [SD] age, 63.5 [14.8] years; 2437 female [51.0%] and 2342 male [49.0%]) had a palliative care consultation with a PPS score documented. Of these patients, 2276 were seen in the inpatient setting and 3080 were seen in the outpatient setting. In both the inpatient and outpatient settings, 1-, 6-, and 12-month mortality were higher and median survival was shorter for patients with lower PPS scores. Prognostic estimates associated with PPS scores were substantially longer (2.3- to 11.7-fold) than previous estimates commonly used by clinicians. The PPS had good ability to discriminate between patients who lived and those who died in the inpatient setting (integrated time-dependent area under the curve [iAUC], 0.74) but its discriminative ability was lower in the outpatient setting (iAUC, 0.67). The PPS better predicted 1-month survival than longer-term survival. Mortality rates were higher for patients with cancer than other serious illnesses at most PPS levels.

**Conclusions and Relevance:**

In this prognostic study, prognostic estimates associated with PPS scores were substantially longer than previous estimates commonly used by clinicians. Based on these findings, an online calculator was updated to assist clinicians in reaching prognostic estimates that are more consistent with modern palliative care practice and specific to the patient’s setting and diagnosis group.

## Key Points

**Question:**

What prognoses are associated with various Palliative Performance Scale (PPS) scores in contemporary palliative care practice?

**Findings:**

In this prognostic study of 4479 patients, updated prognostic estimates associated with PPS scores were substantially longer than previous estimates commonly used by clinicians. The PPS better predicted 1-month survival than longer-term survival, and mortality rates for most PPS scores were higher for patients with cancer than other serious illnesses.

**Meaning:**

These findings suggest that the prognosis associated with a PPS score is dependent on the practice setting and the patient’s illness and that clinicians should use modern data when estimating prognoses from PPS scores.

## Teaser

This prognostic study assessed associations of scores on the Palliative Performance Scale with short- and long-term survival to provide updated prognosis estimates for patients with cancer and other serious illnesses.

## Introduction

The ability to prognosticate is key when caring for patients with serious illness. Prognostic information impacts the decisions that people make about their care and many other aspects of their lives.^1^ Knowing what to expect in the future is also important to the family members of people with serious illness.^2^ Prognostication is critical for clinicians considering which patients are eligible for hospice care, the risk-benefit ratio of many treatments, deprescribing preventive medications, and the urgency with which to engage patients and family members in goals of care conversations. However, prognostication is challenging, and doctors are known to be inaccurate in their estimations of prognosis.^3,4^

The Palliative Performance Scale (PPS) is a widely used tool to help clinicians with prognostication for their patients with serious illness, and it is the most frequently used tool on ePrognosis, an online compendium of prognostic tools, where it had 12 405 views from 3032 users in the first 30 days of 2024.^5^ The PPS is a measure of functional status modeled off the Karnofsky Performance Scale and was adapted to be appropriate for seriously ill populations.^6^ The PPS was developed in Canada in the 1990s in home-based and inpatient palliative care units.^6^ A number of studies^6,7,8,9,10,11,12,13,14,15,16,17,18,19,20,21,22,23^ have reported prognoses associated with various PPS scores to aid practicing clinicians. The survival times by PPS score for inpatients shown on the ePrognosis website were derived from a 2011 study^23^ of 958 hospitalized patients (776 [81%] with cancer) referred for a palliative care consult. The median survival of patients in the study was 35 days (95% CI, 31-39 days).^23^ The survival times by PPS for outpatients provided on ePrognosis were derived from a 2014 Canadian study^7^ of 1655 outpatients with cancer seen in a palliative care clinic. The longest survival time in this study was 221 days and was associated with a PPS score of 80% to 100%. A large study of 118 532 hospice patients in the US^17^ also reported the probability of 6-month mortality by PPS. Another large study using data from 11 342 patients seen in a cancer center in Canada between 2007 and 2009,^22^ examined how the hazard of death trended based on PPS score; this is one of the very few studies that reported on the use of PPS in patients with prognoses of more than 1 year.

The preponderance of prior literature examining prognoses associated with PPS scores is from hospice or inpatient palliative care settings in which prognoses were generally short and is based on data from over 10 years ago. Additionally, the majority of outpatient studies focused only on patients with cancer. The practice of palliative care has changed substantially since the advent of the PPS in the 1990s. Palliative care clinicians are now seeing many more patients upstream of the end-of-life period, in the outpatient setting (including by video telemedicine), and with a wider range of noncancer diagnoses. Furthermore, the accuracy of the PPS at predicting short-term (eg, 1 month) vs long-term (eg, 1 year) survival has not been rigorously evaluated. We sought to address these gaps by examining prognoses associated with various PPS scores in patients seen by inpatient and outpatient specialty palliative care at a large academic medical center in the US. We conducted an external validation to evaluate the performance of the PPS in inpatient and outpatient settings across a range of survival times and among subgroups of interest (people with cancer vs other serious illnesses and those who are seen for in-person vs video telemedicine outpatient visits) to provide updated and more specific prognostic guidance for clinicians and, in turn, patients.

## Methods

### Setting and Patient Population

This prognostic study was approved by the institutional review board of University of California, San Francisco and followed the Transparent Reporting of a Multivariable Prediction Model for Individual Prognosis or Diagnosis (TRIPOD) reporting guideline.^29^ The study used data from the electronic health record (EHR) of patients seen by a specialty palliative care team in the inpatient or outpatient setting at University of California, San Francisco. We included patients seen for at least 1 palliative care encounter between January 1, 2018, and December 31, 2020, in which a PPS score was documented; patients were counted more than once if seen for both inpatient and outpatient encounters. The institutional review board granted an exemption for informed consent because this study used only retrospective data from the EHR and California Vital Records registry. We excluded patients who lived outside California because death information was not available for them. No patients were excluded due to loss to follow-up because California Vital Data includes date of death for all California residents known to have died, including those who died out of state.^24^

### Measures

The PPS was recorded in each templated initial palliative care note in the inpatient and outpatient settings, as well as in follow-up palliative care notes in the outpatient setting.^6^ PPS scores range from 0% to 100% in increments of 10% with 0% indicating deceased, 10% indicating worst functional status, and 100% indicating best functional status. For example, at a PPS score of 70%, a person has reduced ambulation and cannot do usual work activities but is still able to care for themself and has normal oral intake and consciousness, whereas at a PPS score of 30%, a person is bed bound, requires total care, and has reduced (but not minimal) oral intake. The score was determined by the palliative care physician completing the note. A description of each PPS score was included in the note template to assist physicians in selecting the most appropriate PPS score (eFigure 1 in Supplement 1).

Demographic characteristics extracted from the EHR, including sex and race and ethnicity, were determined by patient self-report on a standardized form at the time of the patient’s initial visit in the health system. We reported the race and ethnicity of our sample to characterize the diversity of the study population. Race and ethnicity categories included Asian, Black, Hispanic, Native Hawaiian or Other Pacific Islander, multiracial, unknown, or other (defined as any race or ethnicity not otherwise specified). Categories are listed in the specific way that they were collected from patients and stored in the EHR database with the exception that we combined separate questions on race and ethnicity and categorized patients as Latine when the patient reported Hispanic ethnicity.

### Procedure

Demographics and clinical characteristics were electronically abstracted from the EHR. To examine the association of PPS score with 1-month, 6-month, and 12-month mortality as well as median survival, we used the PPS score from the first palliative care encounter in the setting. Whether patients died within 30 months of follow-up and date of death were determined by linking information in EHR with California Vital Records. Serious illness diagnosis category (cancer or other) was determined based on the primary diagnosis listed in the templated initial palliative care consult note. Location (inpatient vs outpatient) and mode of the visit (in-person vs video telemedicine) at the time of the PPS score were obtained from the EHR electronically. Patients were excluded from the mode of visit analysis if the mode of the initial palliative case visit was not known.

### Statistical Analysis

Descriptive statistics were used to summarize demographic and clinical characteristics of the cohort. We examined the association of PPS score with 1-month, 6-month, and 12-month mortality using bivariate analyses and median survival (truncated at 30 months of follow-up) with 95% CIs using Kaplan-Meier curves. To determine predicted median time to death with 95% CIs, we conducted parametric survival analyses using a Weibull proportional hazard model and we calculated hazard ratios after adjusting for age, gender, and diagnosis group. Performance of the PPS across a range of survival times was examined using discrimination, a measure of a prognostic model’s ability to differentiate those who lived from those who died. We calculated time-specific area under the receiver operating characteristic curve (AUC) values at 1, 6, and 12 months, as well as integrated time-dependent AUC (iAUC) values, which average all available AUC statistics over time.^25,26,27^ We assessed internal validity via bootstrapping to quantify any optimism in model performance. We repeated the entire modeling process in 100 bootstrapped samples and obtained the model optimism, which was defined as averaged difference between apparent performance and original dataset performance.^28^ We calculated optimism-corrected iAUC and AUC for inpatient and outpatient settings separately. Subgroup analyses were performed for diagnosis category (cancer vs noncancer) and by visit mode (in-person vs video) for patients seen in the outpatient setting. An α < .05 was used to determine statistical significance.

Statistical analyses were conducted using SAS version 9.4 (SAS Institute) and Stata version 17 (Stata Corp). Data analysis was conducted from November 2022 to February 2024.

## Results

During the study period, 4779 patients (mean [SD] age, 63.5 [14.8] years; 2437 female [51.0%] and 2342 male [49.0%]; 801 Asian [16.8%]; 631 Latine [13.2%]; 2737 White [57.3%]; 4122 who preferred English [86.3%]) had a palliative care consultation in which a PPS score was documented and were included in our study cohort (Table 1). Most were married and had Medicare. Nearly one-half of the patient encounters (2276 encounters [47.6%]) were inpatient palliative care consultations and nearly two-thirds of the patient encounters (3080 encounters [64.4%]) were outpatient palliative care consultations. In the inpatient setting, there were 1419 patient encounters (62.3%) with cancer as the serious illness leading to palliative care referral, while in the outpatient setting, there were 2277 patient encounters (73.9%) with cancer as the serious illness leading to palliative care referral. A total of 14 clinicians who gave PPS scores practiced in the inpatient setting and 19 clinicians practiced in the outpatient palliative care setting.

**Table 1.** Demographic and Clinical Characteristics

Characteristics	Participants, No. (%)		
	Overall population (N = 4779)	Inpatients (n = 2276)	Outpatients (n = 3080)
Age, mean (SD) [range], y	63.5 (14.8) [16-100]	62.1 (15.6) [16-100]	63.7 (14.4) [18-100]
Age category, y			
<45	577 (12.1)	335 (14.7)	352 (11.4)
45-55	638 (13.4)	342 (15.0)	391 (12.7)
55-65	1177 (24.6)	552 (24.3)	767 (24.9)
65-75	1352 (28.3)	591 (26.0)	914 (29.7)
75-85	753 (15.8)	313 (13.8)	497 (16.1)
>85	282 (5.9)	143 (6.3)	159 (5.2)
Sex			
Female	2437 (51.0)	1172 (51.5)	1592 (51.7)
Male	2341 (49.0)	1104 (48.5)	1487 (48.3)
Non-binary	1 (<0.1)	0	1 (<0.1)
Race and ethnicity			
American Indian or Alaska Native	26 (0.5)	17 (0.7)	11 (0.4)
Asian	801 (16.8)	411 (18.1)	490 (15.9)
Black	295 (6.2)	189 (8.3)	143 (4.6)
Latine	631 (13.2)	350 (15.4)	373 (12.1)
Multiracial	102 (2.1)	45 (2.0)	71 (2.3)
Native Hawaiian or Other Pacific Islander	31 (0.6)	16 (0.7)	17 (0.6)
White	2737 (57.3)	1166 (51.2)	1891 (61.4)
Other^a^	102 (2.1)	53 (2.5)	58 (1.8)
Unknown	54 (1.1)	29 (1.3)	29 (0.8)
Language			
English	4122 (86.3)	1889 (83.0)	2735 (88.8)
Chinese	263 (5.5)	152 (6.7)	140 (4.5)
Spanish	204 (4.3)	131 (5.8)	100 (3.2)
Other	190 (3.9)	104 (4.6)	105 (3.4)
Partnered status			
Single	1095 (22.9)	601 (26.4)	637 (20.7)
Married or partnered	2722 (57)	1227 (53.9)	1844 (59.9)
Divorced or separated	448 (9.4)	222 (9.8)	275 (8.9)
Widowed	374 (7.8)	178 (7.8)	227 (7.4)
Unknown or declined	140 (2.9)	48 (2.1)	97 (3.1)
Insurance status			
Medicare	2742 (57.4)	1198 (52.6)	1821 (59.1)
Medicaid	774 (16.2)	503 (22.1)	383 (12.4)
Private or other	1263 (26.4)	575 (25.3)	876 (28.4)
Access to electronic health record patient portal	1860 (38.9)	536 (23.6)	1489 (48.3)
Serious illness diagnosis group			
Cancer	3203 (67.0)	1419 (62.3)	2277 (73.9)
Neurologic	475 (9.9)	116 (5.1)	365 (11.9)
Other	1101 (23.1)	741 (32.6)	438 (14.2)

^a^ Other was defined as any other race or ethnicity not otherwise specified.

At the end of 30-month follow-up, 1393 patients seen in the inpatient palliative care setting (61.2%) had died and 1346 seen in the outpatient palliative care setting (43.7%) had died. One-month, 6-month, and 12-month mortality as well as median survival for various PPS scores in the inpatient and outpatient settings are shown in Table 2.

**Table 2.** Palliative Performance Scale Score and 1-Month, 6-Month, and 12-Month Mortality and Median Survival

Palliative Performance Scale score, %	Mortality, No. (%) (N = 4779)^a^			Survival, median (95% CI), mo
	1-mo	6-mo	12-mo	
Inpatient setting (n = 2276)				
10 (n = 218)	144 (66.1)	173 (79.4)	174 (79.8)	0.59 (0.46 to 0.72)
20 (n = 157)	92 (58.6)	114 (72.6)	117 (74.5)	0.69 (0.53 to 0.92)
30 (n = 277)	121 (43.7)	177 (63.9)	188 (67.9)	1.51 (1.02 to 2.00)
40 (n = 391)	123 (31.5)	220 (56.3)	255 (65.2)	2.83 (2.04 to 4.86)
50 (n = 389)	84 (21.6)	179 (46.0)	201 (51.7)	9.79 (5.59 to 19.29)
60 (n = 429)	52 (12.1)	161 (37.5)	191 (44.5)	21.85 (12.98 to ≥30.00)
70 (n = 293)	28 (9.6)	93 (31.7)	120 (41.0)	≥30.00 (16.72 to ≥30.00)
80-100 (n = 122)	6 (4.9)	27 (22.1)	39 (32.0)	≥30.00 (28.45 to ≥30.00)
Outpatient setting (n = 3080)				
10-30 (n = 72)	17 (23.6)	30 (41.7)	40 (55.6)	8.02 (5.32 to ≥30.00)
40 (n = 86)	14 (16.3)	34 (39.5)	39 (45.4)	21.16 (5.68 to ≥30.00)
50 (n = 273)	34 (12.5)	88 (32.2)	118 (43.2)	18.99 (12.45 to 28.62)
60 (n = 553)	34 (6.2)	174 (31.5)	244 (44.1)	17.64 (12.98 to 23.75)
70 (n = 1050)	36 (3.4)	220 (21.0)	347 (33.0)	≥30.00 (≥30.00 to ≥30.00)
80-100 (n = 1046)	7 (0.7)	106 (10.1)	185 (17.7)	≥30.00 (≥30.00 to ≥30.00)

^a^ Percentages by row.

Figure, A shows survival rates over time for inpatients. Mortality rate was highest in the least functional PPS groups, and more than 50% of patients with PPS scores of 10% and 20% died within 1 month of when the PPS was measured (PPS 10%, 144 of 218 patients [66.1%]; PPS 20%, 92 of 157 patients [58.6%]). The mortality rate was lower in more functional PPS groups, with fewer than 50% of patients (460 of 1233 patients [37.3%]) dying by 6 months when PPS was greater than 50%. In all PPS groups, mortality was highest within the first month after PPS was measured, and by 12 months of follow-up, the mortality rate in all PPS groups was low and similar. Figure, B shows survival rates over time for outpatients. Again, mortality rate was greatest in the lower PPS groups early on, but the survival rate was similar between PPS groups after 12 months of follow-up. The ability of PPS to discriminate between patients who lived and patients who died was good overall in the inpatient setting (iAUC, 0.74) and better at 1 month (AUC, 0.76) than at 6 months (AUC, 0.68) and 12 months (AUC, 0.66). In the outpatient setting, the ability of the PPS to discriminate between patients who lived and died was lower overall (iAUC, 0.67) but, like the inpatient setting, it was better at 1 month (AUC, 0.76). Internal bootstrapping demonstrated an optimism correction of less than .01 for iAUC and time-specific AUC in the inpatient and outpatient settings, suggesting minimal overfitting. AUCs across a range of follow-up time intervals are shown in eFigure 2 in Supplement 1.

**Figure.**
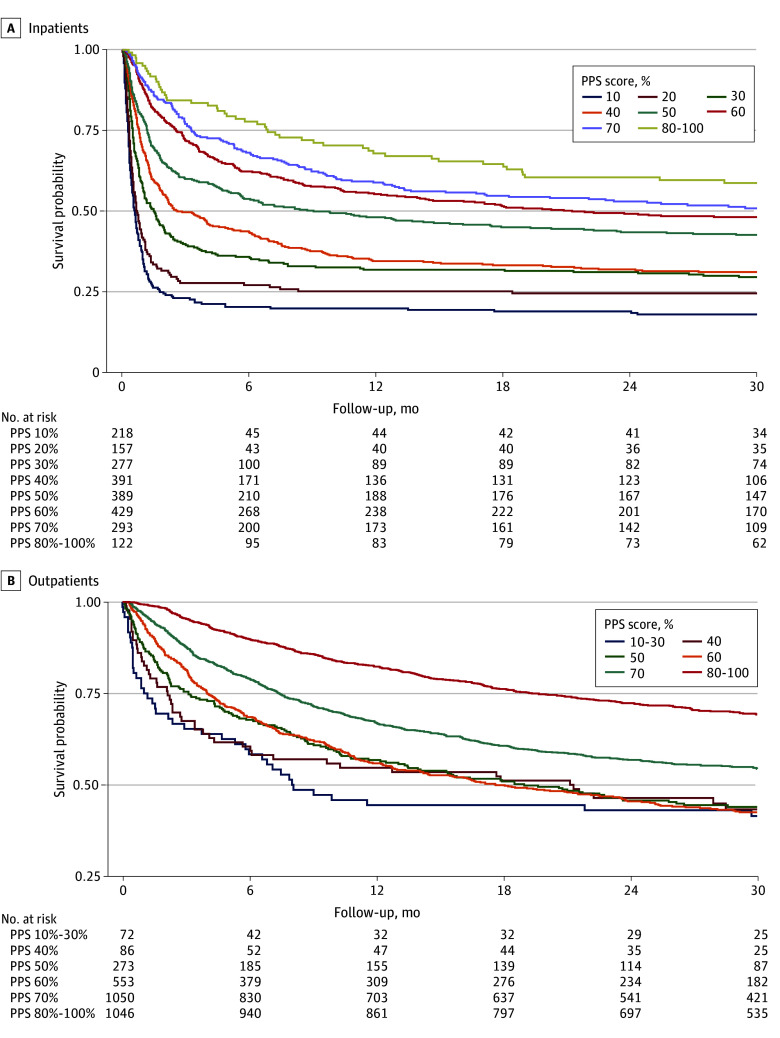
Probability of Survival Over Time by Palliative Performance Scale (PPS) Score

In the inpatient setting, iAUC did not change with the addition of age, sex, and diagnosis group to the model (0.74 vs 0.74), but in the outpatient setting, adding diagnosis group to the model increased the iAUC slightly (0.67 vs 0.70). The hazard ratios and median survivals are shown in eTable 1 in Supplement 1.

For patients seen by palliative care in the outpatient setting, 1680 (54.7%) had their initial visit in-person and 1393 (45.3%) had their initial visit by video telemedicine. There was no significant difference between median survival by PPS score for people seen in-person and by video.

In both the inpatient and outpatient settings, mortality rates were higher for patients with cancer at most PPS levels than for patients with other serious illnesses (Table 3). This difference was particularly pronounced in the outpatient setting.

**Table 3.** Prognosis Associated With PPS Score by Disease Category

PPS score, %	Cancer				PPS score,%^b^	Noncancer serious illness			
	Mortality^a^			Survival, median (95% CI), mo		Mortality^a^			Survival, median (95% CI) survival, mo
	Total No.	1-mo, No. (%)	6-mo, No. (%)			Total No.	1-mo, No. (%)	6-mo, No. (%)	
**Inpatient** ^b^									
10	48	31 (64.6)	41 (85.4)	0.39 (0.26 to 0.82)	10	170	113 (66.5)	132 (77.7)	0.62 (0.49 to 0.76)
20	68	41 (60.3)	49 (72.1)	0.59 (0.43 to 1.08)	20	89	51 (57.3)	65 (73.0)	0.75 (0.53 to 1.08)
30	139	65 (46.8)	97 (69.8)	1.31 (0.85 to 1.97)	30	138	56 (40.6)	80 (58.0)	1.68 (1.05 to 6.08)
40	233	75 (32.2)	131 (56.2)	3.29 (1.87 to 6.14)	40	158	48 (30.4)	89 (56.3)	2.30 (1.77 to 6.11)
50	268	67 (25.0)	142 (53.0)	4.70 (2.33 to 8.15)	50	121	17 (14.1)	37 (30.6)	≥30.00 (22.28 to ≥30)
60	339	44 (13.0)	135 (39.8)	17.87 (10.19 to ≥30.00)	60	90	8 (8.9)	26 (28.9)	≥30.00 (14.49 to ≥30.00)
70	229	24 (10.5)	77 (33.6)	22.83 (10.11 to ≥30.00)	70	64	4 (6.3)	16 (25.0)	≥30.00 (≥30.00 to ≥30.00)
80-100	95	6 (6.3)	22 (23.2)	≥30.00 (18.89 to ≥30.00)	80-100	27	0 (0.0)	5 (18.5)	≥30.00 (14.95 to ≥30.00)
**Outpatient** ^c^									
10-30	17	9 (52.9)	12 (70.6)	0.89 (0.26 to 7.89)	10-30	55	8 (14.6)	18 (32.7)	21.85 (6.83 to ≥30.00)
40	38	9 (23.7)	21 (55.3)	3.42 (1.64 to 27.93)	40	48	5 (10.4)	13 (27.1)	≥30.00 (17.68 to ≥30.00)
50	134	29 (21.6)	58 (43.3)	9.99 (4.70 to 20.63)	50	139	5 (3.6)	30 (21.6)	28.62 (17.87 to ≥30.00)
60	356	28 (7.9)	140 (39.3)	10.71 (8.35 to 14.46)	60	197	6 (3.1)	34 (17.3)	≥30.00 (23.79 to ≥30.00)
70	839	35 (4.2)	198 (23.6)	≥30.00 (23.49 to ≥30.00)	70	211	1 (0.5)	22 (10.4)	≥30.00 (≥30.00 to ≥30.00)
80-100	893	7 (0.8)	99 (11.1)	≥30.00 (≥30.00 to ≥30.00)	80-100	153	0	7 (4.6)	≥30.00 (≥30.00 to ≥30.00)

Abbreviation: PPS, Palliative Performance Scale.

^a^ Percentages by row.

^b^ The participant totals for inpatients were 1419 patients with cancer, and 857 patients with noncancer serious illness.

^c^ The participant totals for outpatients were 2277 patients with cancer, and 803 patients with noncancer serious illness.

Overall, we found substantially longer prognoses associated with PPS scores than most earlier studies,^6,18,19,20^ including those that were previously used to generate prognostic estimates on ePrognosis (eTable 2 in Supplement 1). For example, we found that an inpatient with a PPS of 50% had a prognosis of 298 days, which differs substantially from the estimate currently provided on ePrognosis of 54 days (derived from Jang et al^7^). In fact, we found median survival measurements by PPS score that were 2.3-11.7 times longer than those previously used to generate the estimates shown on ePrognosis.^7,23^

## Discussion

In this external validation prognostic study using data from a large academic medical center with robust inpatient and outpatient palliative care services serving patients with a wide range of serious illnesses, we generated updated estimates of the prognoses associated with various PPS scores in seriously ill populations, and examined how the predictive ability of the PPS varies by prognostic time frame. We also explored how prognoses vary by diagnosis group and mode of visit.

Like prior studies,^6,18,19,20^ our data demonstrate that setting matters; for the same level of disability as measured by the PPS, prognoses were substantially longer for people with serious illness seen in the outpatient setting compared with the inpatient setting. However, we found substantially longer prognoses associated with PPS scores than most earlier studies,^6,18,19,20^ including those that were previously used to generate prognostic estimates on ePrognosis (eTable 2 in Supplement 1). In both the inpatient and outpatient settings, many patients in modern palliative care practices are seen earlier in their disease course than they were in the past. These patients are not imminently dying and may live with substantial debility for months or years. Additionally, PPS scores correlated with shorter prognoses in patients with cancer compared with patients with other serious illnesses, particularly in the outpatient setting. It is therefore important for clinicians to use prognostic estimates that were derived in a setting and population similar to theirs. We have used our results to update the calculator on the ePrognosis website so that clinicians can reach prognostic estimates based on modern data specific to their patient’s care setting and diagnosis group.^5^ In addition to displaying median survival, we added 1- and 6-month mortality estimates.

In the inpatient palliative care setting, the PPS had good discriminative power particularly for estimating short-term (eg, 1-month) mortality, which is often an important focus in that setting. However, in the outpatient setting the PPS had only moderate predictive value and should not be relied upon alone without also considering other factors including the patient’s specific diagnosis, disease trajectory, and the clinician’s judgement. This is not to say that the PPS is unimportant in this setting, particularly because the PPS is not only a prognostic tool but also a measure of a patient’s functional status, which can help identify patients who need various services and supports. However, it may be that using disease-specific prognostic tools, such as the Bode Index^30^ for chronic obstructive pulmonary disease, the Seattle Heart Failure Model,^31^ and the Deardorff model for dementia,^32^ could provide better prognostic estimates in the outpatient setting and when prognosis is long. These tools should be tested in the outpatient palliative care setting. Additionally, regardless of how a clinician arrives at a prognostic estimate, when estimating longer-term mortality and median survival, clinicians must acknowledge the inherent uncertainty in prognostication.

It is reassuring that prognoses associated with PPS scores were similar for patients seen in-person and by video telemedicine in outpatient palliative care. This finding suggests that PPS can be accurately determined through video telemedicine visits, a mode that is increasingly being used to provide outpatient palliative care.^33,34^

### Limitations

Limitations of this study include that it was performed using data from a single health system. However, both the inpatient and outpatient palliative care practices were large—with 14 clinicians practicing in the inpatient and 19 clinicians practicing in the outpatient palliative care setting—and patient populations came from dozens of referring services and from across the state of California, which enhances generalizability. Our population was younger than many other palliative care populations, but this is unlikely to impact findings because age did not significantly impact the association of PPS score with prognosis. Our population was also not racially representative of the US population; however, it is unlikely that this affected our findings because previous literature has shown that the PPS performs similarly in White and racial and ethnic minority populations.^9^ PPS scores were determined by palliative care clinicians in the course of usual clinical care, but note templates (including a description of each PPS score) supported clinicians to choose the appropriate score, which is typical of how the PPS is determined in practice. In both the inpatient and outpatient settings, there were not enough patients within some PPS categories such that we had to collapse categories, limiting the granularity of our findings. Similarly, there were not enough patients to separate diagnoses into more specific groups than cancer and noncancer serious illnesses. Furthermore, there were not enough patients with multiple PPS measurements over time to be able to examine whether PPS trajectory is associated with prognosis. Finally, we had only 30 months of follow-up on patients, so we had to truncate our findings at this point. A future study using a larger, multisite dataset that includes multiple PPS measurements over a longer period of follow-up could allow for more specific prognostic estimates for groups with long prognosis and evaluate whether information about PPS trajectory could improve prognostic accuracy.

## Conclusions

In conclusion, we determined prognoses were associated with PPS scores in contemporary inpatient and outpatient palliative care services at a large academic medical center. We found that the ability of the PPS to predict mortality was better in the inpatient setting and for short-term mortality, and that the setting of care and disease category impact prognostic estimates. We have used our results to update the calculator on the ePrognosis website so that clinicians can get prognostic estimates based on modern data specific to their patient’s care setting and diagnosis group. Further study is needed to better elucidate whether PPS trajectory can add precision to prognostic estimates and to compare the performance of the PPS with disease specific prognostic tools in the outpatient palliative care setting.

## Notes

Supplement 1. **eFigure 1.** Description of Each Palliative Performance Scale (PPS) Score as Described in the Note Template **eFigure 2**. Receiver Operating Characteristic (ROC) Curve for PPS Score and Survival, Area Under the Curve (AUC) and Integrated Time-Dependent Area Under the Curve (iAUC) by Setting **eTable 1.** Association Between PPS Score and Median Survival **eTable 2.** Comparison With Prior Published Data

Supplement 2.
Data Sharing Statement
