# Supplementary material for: Prognoses Associated With Palliative Performance Scale Scores in Modern Palliative Care Practice
Source: JAMA Netw Open. 2024 Jul 8;7(7):e2420472. doi: 10.1001/jamanetworkopen.2024.20472 (PMC11231792; doi:10.1001/jamanetworkopen.2024.20472)
Supplement: Supplement 1. — eFigure 1. Description of Each Palliative Performance Scale (PPS) Score as Described in the Note Template eFigure 2. Receiver Operating Characteristic (ROC) Curve for PPS Score and Survival, Area Under the Curve (AUC) and Integrated Time-Dependent Area Under the Curve (iAUC) by Setting eTable 1. Association Between PPS Score and Median Survival eTable 2. Comparison With Prior Published Data [file jamanetwopen-e2420472-s001.pdf]

# Supplemental Online Content

Bischoff KE, Patel K, Boscardin WJ, O'Riordan DL, Pantilat SZ, Smith AK. Prognoses associated with Palliative Performance Scale scores in moden palliative care practice. *JAMA Netw. Open*. 2024;7(7):e2420472. doi:10.1001/jamanetworkopen.2024.20472

**eFigure 1.** Description of Each Palliative Performance Scale (PPS) Score as Described in the Note Template

**eFigure 2.** Receiver Operating Characteristic (ROC) Curve for PPS Score and Survival, Area Under the Curve (AUC) and Integrated Time-Dependent Area Under the Curve (iAUC) by Setting

**eTable 1.** Association Between PPS Score and Median Survival

**eTable 2.** Comparison With Prior Published Data

This supplemental material has been provided by the authors to give readers additional information about their work.

© 2024 Bischoff KE et al. *JAMA Network Open.*

## eFigure 1. Description of Each Palliative Performance Scale (PPS) Score as Described in the Note Template

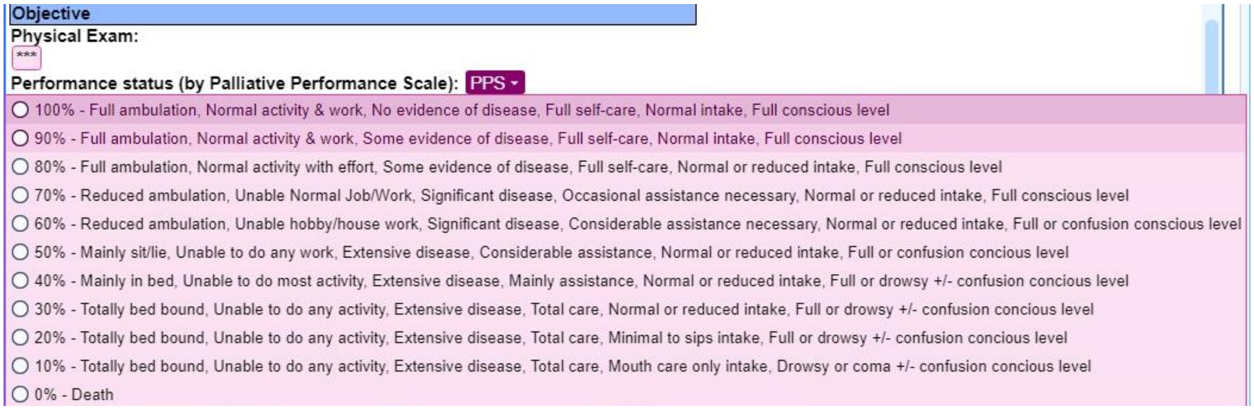

Objective

Physical Exam:

***

Performance status (by Palliative Performance Scale): PPS

- 100% - Full ambulation, Normal activity & work, No evidence of disease, Full self-care, Normal intake, Full conscious level
- 90% - Full ambulation, Normal activity & work, Some evidence of disease, Full self-care, Normal intake, Full conscious level
- 80% - Full ambulation, Normal activity with effort, Some evidence of disease, Full self-care, Normal or reduced intake, Full conscious level
- 70% - Reduced ambulation, Unable Normal Job/Work, Significant disease, Occasional assistance necessary, Normal or reduced intake, Full conscious level
- 60% - Reduced ambulation, Unable hobby/house work, Significant disease, Considerable assistance necessary, Normal or reduced intake, Full or confusion conscious level
- 50% - Mainly sit/lie, Unable to do any work, Extensive disease, Considerable assistance, Normal or reduced intake, Full or confusion concious level
- 40% - Mainly in bed, Unable to do most activity, Extensive disease, Mainly assistance, Normal or reduced intake, Full or drowsy +/- confusion concious level
- 30% - Totally bed bound, Unable to do any activity, Extensive disease, Total care, Normal or reduced intake, Full or drowsy +/- confusion concious level
- 20% - Totally bed bound, Unable to do any activity, Extensive disease, Total care, Minimal to sips intake, Full or drowsy +/- confusion concious level
- 10% - Totally bed bound, Unable to do any activity, Extensive disease, Total care, Mouth care only intake, Drowsy or coma +/- confusion concious level
- 0% - Death

© 2024 Bischoff KE et al. *JAMA Network Open.*

**Supplemental Figure 2. Receiver Operating Characteristic (ROC) Curve for PPS Score and Survival, Area Under the Curve (AUC) and Integrated Time-Dependent Area Under the Curve (iAUC) by Setting**

eFigure 2a: ROC curve, AUC and iAUC, unadjusted model in inpatient setting

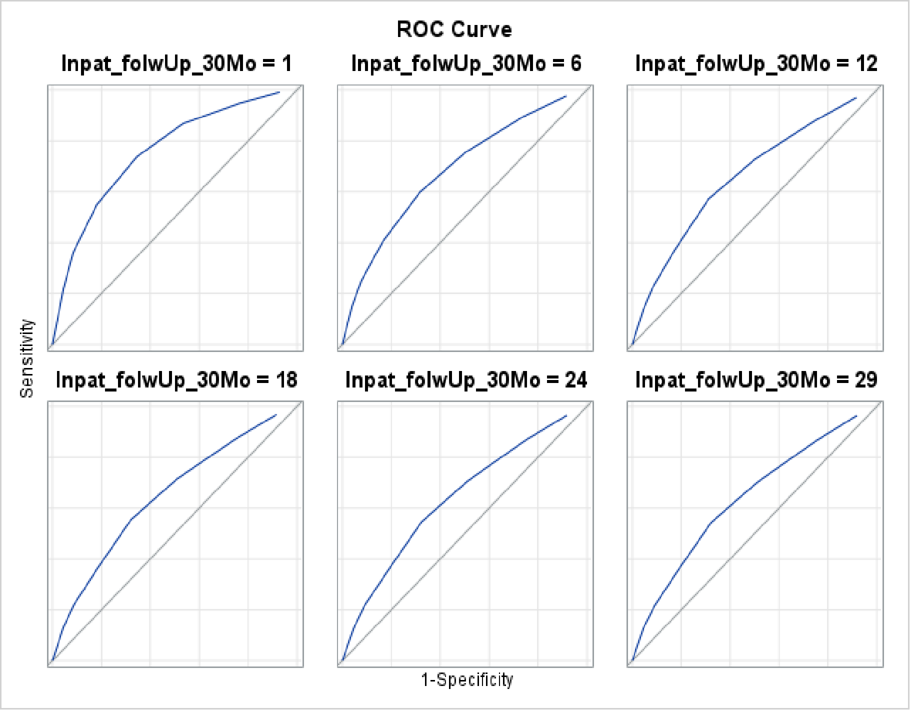


eFigure 2b: ROC curve, AUC and iAUC, addition of age, sex and diagnosis in unadjusted model in inpatient setting

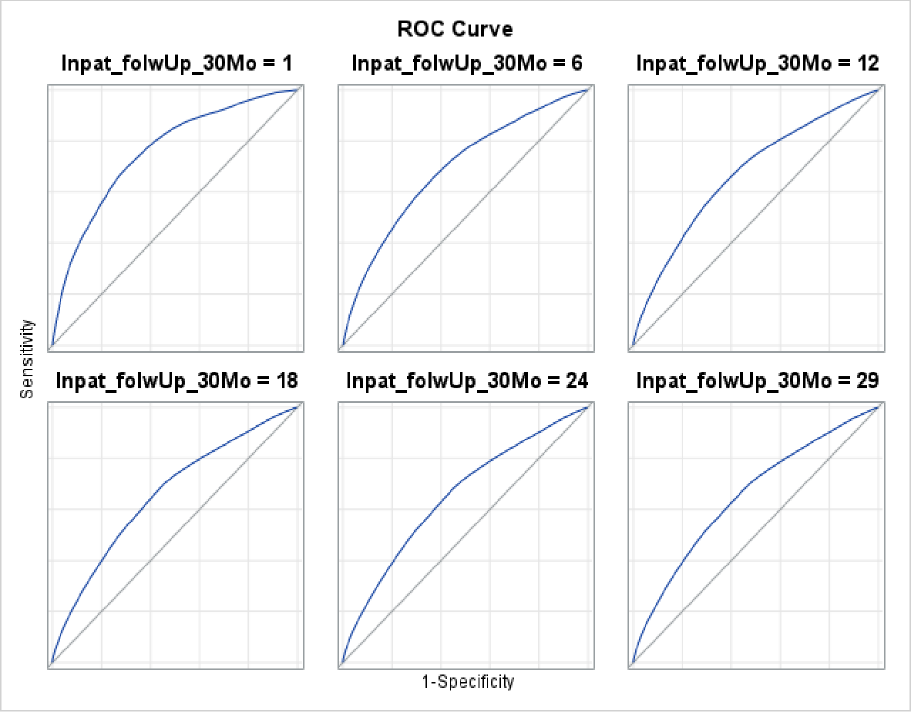


© 2024 Bischoff KE et al. *JAMA Network Open.*

eFigure 2c: ROC curve, AUC and iAUC, unadjusted model in outpatient setting

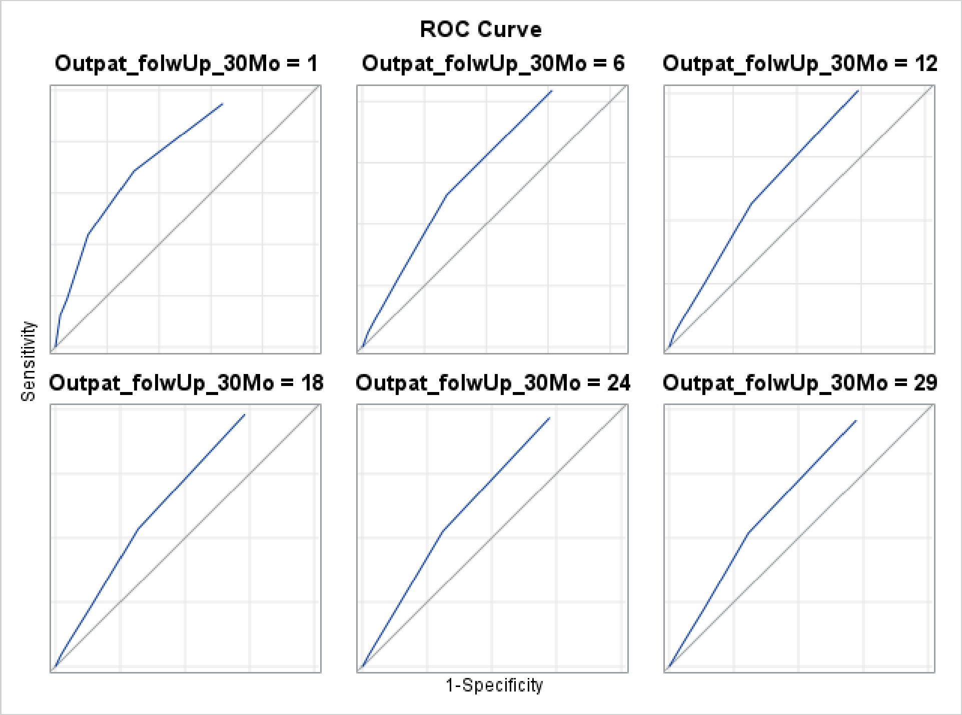


eFigure 2d: ROC curve, AUC and iAUC, addition of age, sex and diagnosis in unadjusted model in outpatient setting

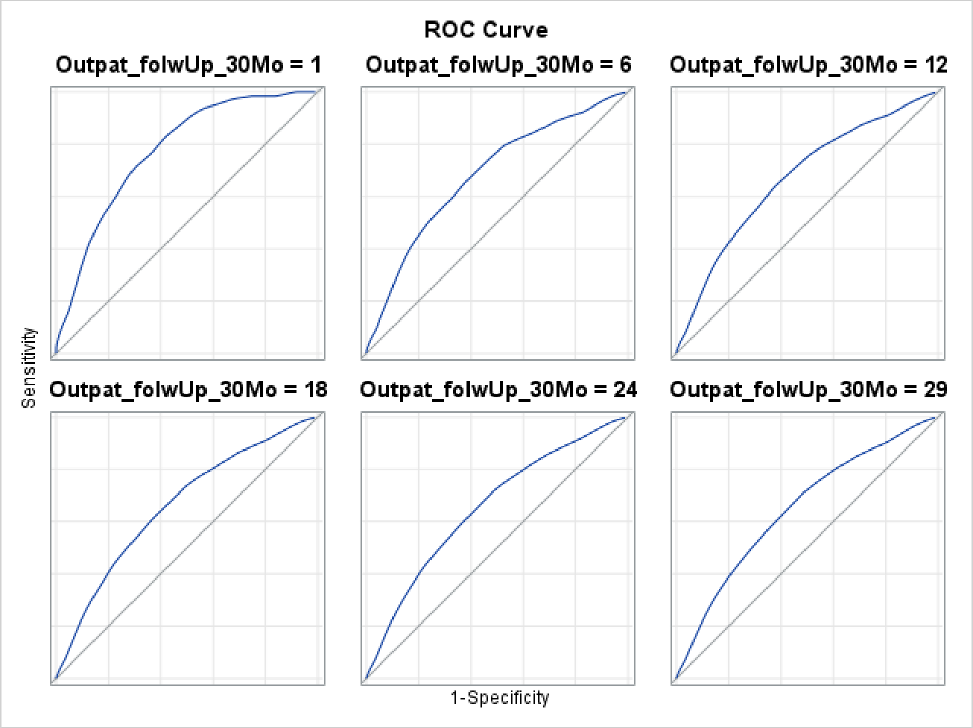


© 2024 Bischoff KE et al. *JAMA Network Open.*

## eTable 1. Association Between PPS Score and Median Survival

| **Inpatient setting (n=2276)** | | | |
|---|---|---|---|
| PPS | Hazard ratio (95% CI), **unadjusted** | Hazard ratio (95% CI), **age-sex adjusted** | Hazard ratio (95% CI), **age-sex-diagnosis adjusted** |
| 10% (n=218) | 4.72 (3.45 - 6.47); p≤0.0001 | 4.34 (3.16 - 5.95); p≤0.0001 | 5.06 (3.66 - 6.99); p≤0.0001 |
| 20% (n=157) | 3.73 (2.68 - 5.20); p≤0.0001 | 3.33 (2.39 - 4.66); p≤0.0001 | 3.64 (2.60 - 5.10); p≤0.0001 |
| 30% (n=277) | 2.86 (2.10 - 3.91); p≤0.0001 | 2.63 (1.93 - 3.60); p≤0.0001 | 2.86 (2.08 - 3.91); p≤0.0001 |
| 40% (n=391) | 2.53 (1.87 - 3.42); p≤0.0001 | 2.28 (1.68 - 3.09); p≤0.0001 | 2.40 (1.77 - 3.25); p≤0.0001 |
| 50% (n=389) | 1.78 (1.31 - 2.42); p=0.0004 | 1.65 (1.21 - 2.24); p=0.002 | 1.71 (1.25 - 2.32); p=0.001 |
| 60% (n=429) | 1.44 (1.06 - 1.96); p=0.020 | 1.37 (1.01 - 1.87); p=0.044 | 1.37 (1.01 - 1.87); p=0.044 |
| 70% (n=293) | 1.30 (0.94 - 1.79); p=0.112 | 1.27 (0.92 - 1.76); p=0.140 | 1.28 (0.93 - 1.77); p=0.135 |
| 80-100% (n=122) | Ref | Ref | Ref |
| PPS | Median survival (95% CI) in months, **unadjusted** | Median survival (95% CI) in months, **age-sex adjusted** | Median survival (95% CI) in months, **age-sex-diagnosis adjusted** |
| 10% (n=218) | 1.71 (1.13 - 2.28) | 1.90 (1.25 - 2.54) | 1.56 (1.02 - 2.09) |
| 20% (n=157) | 2.90 (1.71 - 4.08) | 3.41 (1.99 - 4.84) | 3.23 (1.89 - 4.58) |
| 30% (n=277) | 5.25 (3.58 - 6.93) | 5.79 (3.92 - 7.66) | 5.57 (3.78 - 7.36) |
| 40% (n=391) | 6.96 (5.07 - 8.84) | 7.98 (5.73 - 10.22) | 8.23 (5.91 - 10.56) |
| 50% (n=389) | 15.37 (10.82 - 19.91) | 16.56 (11.58 - 21.54) | 17.59 (12.25 - 22.93) |
| 60% (n=429) | 24.66 (17.34 to ≥30) | 24.93 (17.47 to ≥30) | 28.56 (19.72 to ≥30) |
| 70% (n=293) | ≥30 (19.57 to ≥30) | 29.34 (18.49 to ≥30) | ≥30 (20.80 to ≥30) |
| 80-100% (n=122) | ≥30 (21.01 to ≥30) | ≥30 (19.09 to ≥30) | ≥30 (21.63 to ≥30) |
| | | | |
| **Outpatient setting (n=3080)** | | | |
| PPS | Hazard ratio (95% CI), **unadjusted** | Hazard ratio (95% CI), **age-sex adjusted** | Hazard ratio (95% CI), **age-sex-diagnosis adjusted** |
| 10-30% (n=72) | 2.94 (2.13 - 4.06); p≤0.0001 | 2.83 (2.05 - 3.91); p≤0.0001 | 4.58 (3.27 - 6.41); p≤0.0001 |
| 40% (n=86) | 2.62 (1.93 - 3.54); p≤0.0001 | 2.51 (1.85 - 3.41); p≤0.0001 | 3.38 (2.48 - 4.60); p≤0.0001 |
| 50% (n=273) | 2.48 (2.04 - 3.01); p≤0.0001 | 2.25 (1.85 - 2.74); p≤0.0001 | 2.89 (2.36 - 3.54); p≤0.0001 |
| 60% (n=553) | 2.49 (2.13 - 2.91); p≤0.0001 | 2.34 (2.00 - 2.74); p≤0.0001 | 2.72 (2.32 - 3.19); p≤0.0001 |
| 70% (n=1050) | 1.74 (1.51 - 2.00); p≤0.0001 | 1.70 (1.47 - 1.96); p≤0.0001 | 1.77 (1.54 - 2.05); p≤0.0001 |
| 80-100% (n=1046) | Ref | Ref | Ref |
| PPS | Median survival (95% CI) in months, **unadjusted** | Median survival (95% CI) in months, **age-sex adjusted** | Median survival (95% CI) in months, **age-sex-diagnosis adjusted** |
| 10-30% (n=72) | 15.78 (8.85 - 22.72) | 16.84 (9.44 - 24.24) | 10.74 (6.03 - 15.45) |
| 40% (n=86) | 18.70 (11.02 - 26.39) | 20.01 (11.79 - 28.23) | 16.61 (9.86 - 23.37) |
| 50% (n=273) | 20.25 (15.58 - 24.93) | 23.49 (17.85 - 29.12) | 20.73 (15.83 - 25.62) |
| 60% (n=553) | 20.07 (16.85 - 23.29) | 22.15 (18.44 - 25.87) | 22.68 (18.81 - 26.55) |
| 70% (n=1050) | ≥30 (29.41 to ≥30) | ≥30 (≥30 to ≥30) | ≥30 (≥30 to ≥30) |
| 80-100% (n=1046) | ≥30 (≥30 to ≥30) | ≥30 (≥30 to ≥30) | ≥30 (≥30 to ≥30) |

*PPS = palliative performance scale, CI = confidence interval

© 2024 Bischoff KE et al. *JAMA Network Open.*

**eTable 2. Comparison With Prior Published Data**

| **Inpatient setting** | | | **Outpatient setting** | | |
|---|---|---|---|---|---|
| PPS | Expected median survival per Tarumi et al. (days)[1] | Observed median survival (days) | PPS | Expected median survival per Jang et al. (days)[2] | Observed median survival (days) |
| 10% | 2 | 18 | 10-30% | 22 | 244 |
| 20% | 5 | 21 | | | |
| 30% | 20 | 46 | 40-50% | 51 | 598 |
| 40% | 31 | 86 | | | |
| 50% | 54 | 298 | 60-70% | 115 | ≥912 |
| 60% | 72 | 665 | | | |
| 70% | 116 | ≥912 | 80-100% | 221 | ≥912 |
| 80% | 121 | ≥912 | | | |
| 90% | 266 | ≥912 | | | |


1. Tarumi Y, Watanabe SM, Lau F, et al. Evaluation of the Palliative Prognostic Score (PaP) and routinely collected clinical data in prognostication of survival for patients referred to a palliative care consultation service in an acute care hospital. *J Pain Symptom Manage*. 2011;42(3):419-431. doi:10.1016/j.jpainsymman.2010.12.013

2. Jang RW, Caraiscos VB, Swami N, et al. Simple prognostic model for patients with advanced cancer based on performance status. *J Oncol Pract*. 2014;10(5):e335-41. doi:10.1200/JOP.2014.001457


© 2024 Bischoff KE et al. *JAMA Network Open.*
