# Supplementary material for: Prognoses Associated With Palliative Performance Scale Scores in Modern Palliative Care Practice
Source: JAMA Netw Open. 2024 Jul 8;7(7):e2420472. doi: 10.1001/jamanetworkopen.2024.20472 (PMC11231792; doi:10.1001/jamanetworkopen.2024.20472)
Supplement: Supplement 2. — Data Sharing Statement [file jamanetwopen-e2420472-s002.pdf]

Data Sharing Statement

Bischoff. Prognoses Associated With Palliative Performance Scale Scores in Modern Palliative Care Practice. *JAMA Netw Open*. Published July 08, 2024.
doi:10.1001/jamanetworkopen.2024.20472

Data
Data available: Yes
Data types: Deidentified participant data
How to access data: kara.bischoff@ucsf.edu
When available: With publication

Supporting Documents
Document types: None

Additional Information
Who can access the data: Anyone requesting the data
Types of analyses: For any purpose
Mechanisms of data availability: With investigator support
Any additional restrictions: None
